# Occupational Health Risks Among Trichloroethylene-Exposed Workers in a Clock Manufacturing Factory

**DOI:** 10.5539/gjhs.v7n1p161

**Published:** 2014-08-22

**Authors:** Siriporn Singthong, Pannee Pakkong, Kantima Choosang, Sarinya Wongsanit

**Affiliations:** 1 Inter Departmental Multidisciplinary Graduate Program in Bioscience, Faculty of Science, Kasetsart University, Bangkok, Thailand; 2 Department of Applied Radiation and Isotope, Faculty of Science, Kasetsart University, Bangkok, Thailand; 3 Faculty of Medical Technology, Rangsit University, Pathumthani, Thailand; 4 Research and Development Group, Thailand Institute of Nuclear Technology, Bangkok, Thailand

**Keywords:** Micronucleus, occupational exposure, trichloroethylene and genotoxicity

## Abstract

Trichloroethylene (TCE) is an important volatile organic compound once widely used in industry throughout the world. Occupational exposure to TCE can cause a number of health hazards such as allergic reactions and genetic damage. The purpose of this study was to evaluate occupational exposure to TCE, by analysis of the air in the breathing zone and of urine from workers employed in a clock manufacturing factory. A subjective symptom survey was conducted by using a self-administered questionnaire to evaluate the health hazards. Micronucleus (MN) frequency, based on the cytokinesis-block micronucleus assay (CBMN) in peripheral blood lymphocytes, (PBLs) was used as a biomarker for chromosome damage. A total of 244 participants, including 171 workers occupationally exposed to TCE and 73 non-exposed control employees, working mainly in office jobs in the same factory, were enrolled in this study. Analyses of airborne TCE concentrations in the workplace, and of urinary trichloroacetic acid (TCA) of the workers and controls, were performed by Gas Chromatography-Electron Capture Detector (GC-ECD) using the modified headspace technique. The average concentration of TCE in the workplace breathing zone was 27.83 ± 6.02 ppm. The average level of urinary TCA of the exposed workers and controls was 14.84 ± 1.62, 2.95 ± 0.28 mg/L. The frequency of MN/1000BN was 7.029 ± 0.39, significantly higher than for those in the control group (3.57 ± 0.31, p = 0.001). According to multiple linear regression analysis, the results indicated that urinary TCA levels correlated with the increased MN in exposed workers (r = 0.285, p < 0.001). The prevalence rate of subjective symptoms in the exposed group was 9.61-11.76 times higher than the rate of the non-exposed group (p < 0.001). It was found that skin (29.6%) and respiratory symptoms (21.1%) were the most frequent among the exposed workers.

In conclusion, these results indicate that increased micronucleus frequency is associated with occupational trichloroethylene exposure. The use of TCE in the factory is threatening workers’ health.

## 1. Introduction

Trichloroethylene (TCE), a volatile organic compound (VOC), is a common industrial solvent used for dry-cleaning and degreasing of fabricated metal parts and has an important application as a lubricant (Agency for Toxic Substances and Disease Registry [ATSDR], 1997). Inhalation is the major route of exposure to trichloroethylene, while the less common routes are ingestion and dermal contact. Consequently, due to its use in many industrial processes, most trichloroethylene is found in the environment as a result of release from factories. TCE has been identified as a major industrial pollutant and environmental contaminant of ground water, surface water and soil (Seo, Kobayashi, & Nagase, 2011; Spencer, Hussein, & Tchounwo, 2006).

TCE is a well-known animal carcinogen (S. Kim, D. Kim, Pollack, Collins, & Rusyn, 2009). It is recognised for its many toxic effects in humans, such as cardiovascular effects, pulmonary toxicity, neurotoxicity and probably genotoxicity. It can cause both acute and chronic toxicity in humans. Some common short-term health effects of TCE exposure are associated with eye, nose, throat, and skin irritation. Those exposed to TCE often report symptoms of fatigue, sleepiness, headache, confusion, and blurred vision (Hansen et al., 2013; Karami et al., 2012; Kumar et al., 2009; Vlaanderen et al., 2013). Although several studies have demonstrated the toxicities of TCE in a variety of systems, genotoxic and carcinogenic effects occurring in humans are still open to question (Scott & Jinot, 2011), even after the US Environment Protection Agency raised TCE’s classification to “human carcinogen” (Kamijima, Hisanaga, Wang, & Nakajima, 2007). Some epidemiological studies have shown that the incidence of urinary-tract tumours and non-Hodgkin lymphomas increased in TCE-exposed workers (Purdue et al., 2011). The toxicity of TCE has focused on the TCE metabolite forms, which are dichloroaceic acid (DCA) and trichloroacetic acid (TCA) (Guha et al., 2012; Moore & Harrington-Brock, 2000). TCA is one of the metabolite forms which is a specific index for TCE absorption. The half life of TCA excretion in urine is about 2 to 5 days. Although TCA levels cannot represent the severity of a TCE-induced disorder, Xu et al. (2009) has shown that they are helpful for diagnosis of symptoms such as dermatitis induced by TCE. The metabolite form of TCE found in the human body may react directly with genetic material, and has also been shown to generate oxidative damage *in vitro*. Long-term exposure to TCE is demonstrated by many severe toxicological and pathological effects such as increased in degeneration or necrosis of hepatocytes and glomerular nephrosis (Karami et al., 2012). TCE exposure has also been shown to induce abnormal DNA synthesis *in vitro* in human lymphocyte, a process that has been associated with increased cancer risk. Occurrences of symptoms that induced by TCE have been reported from many countries such as USA, Japan, Spain, Singapore, China, Korea and Thailand (Kamijima, Hisanaga, Wang, & Nakajima, 2007).

The increased incidences of chromosome abnormalities such as breaks, gaps, deletions and hyperploidy have been observed in lymphocytes of occupationally exposed workers involved in manufacture, and subjects who use TCE (Lavin, Jacobson, & DeSesso, 2000). Cytogenetic biomarkers such as micronucleus assay are one of the preferred methods used to investigate the environment, occupational and medical factors on genomic stability, and are used to evaluate the genotoxic end point in human biomonitors (Ceppi, Biasotti, Fenech, & Bonassi, 2010; El-Zein et al., 2006; Sellappa, Prathyumnan, Joseph, Keyan, & Balachandar, 2010).

A micronucleus (MN) is the small nucleus that arises from acentric chromosome fragments or whole chromosomes not incorporated into one of the daughter cells during mitotic cellular division. Frequency of micronucleus in peripheral blood lymphocytes is accomplished by cytokinesis-block micronucleus assay (CBMN), relying on the observation that cells that have completed nuclear division and have their cytokinesis blocked with cytochalasin B (Fenech, 2007). This method is easier and faster than other popular cytogenetic methods such as metaphase analysis. It can be used either *in vitro* or *in vivo* (Bull et al., 2011; Kirsch-Volders et al., 2011; Sudha, Prathyman, Joseph, & Keyan, 2011).

The purpose of this study was to evaluate urinary TCA and environmental TCE levels as biological markers to determine TCE exposure, and to assess their associations with symptoms of workers in a clock manufacturing factory. Furthermore, this study was also aimed at investigating the DNA damage in TCE-exposed workers, using the cytokinesis blocked micronucleus assay (CBMN).

## 2. Materials and Methods

### 2.1 Population Study

A cross-sectional survey for cytogenetic monitoring was carried out in a clock manufacturing factory, located in Bangkok, Thailand. In this factory, trichloroethylene is used as a degreaser for cleaning the surface metal parts of clocks. The sample size of exposed workers was performed using the formula: n = Z^2^p(1-p)/d^2^, where: Z = Z score for 95% confidence interval = 1.96, p = prevalence, d = tolerable error = 5%. Prevalence of TCE disease in Thailand which reported by Department of Disease control, Ministry of Public Health, is 20%. The estimated dropout rate was 15%.

The study population consisted of 171 workers occupationally exposed to TCE and 73 non-exposed control employees, working mainly in office jobs at the same factory. Each participant of the exposed group and the control group was personally informed of the study aims and interviewed with a standardized questionnaire that included data on age, gender, use of protective equipment, general health status, history of cancer, use of therapeutic drugs, exposure to radiation in the past six months, use of vitamins or other supplements, as well as information related to occupational history, such as working hours per day and years of exposure. All subjects gave informed consent before the study. The Ethical Review Committee for Research, Department of Disease Control approved the study. The characteristics of the exposed subjects and control are shown in Table 1.

**Table 1:** Demographic and physical characteristics among TCE exposed group and control group

Parameters	Exposed (N)	Control (N)	p-value
Total number of individuals	171	73	
Age (years)	35.78±9.54	34.84±12.96	.653
	(18-58)	(18-58)	
Gender			
Male	21.1% (36)	32.8% (24)	
Female	78.9% (135)	67.2% (49)	.057
Duration of work (years)	11.13±10.48	-	-
Hours of work per day	10.77±1.56	-	-
Days of work per week	6.59±0.49	-	-
Use of protective equipments	98.2 % (168)	-	-
Smoking status	8.8% (15)	6.8% (5)	.933
Alcohol consumption	25.1% (43)	31.8% (23)	.201

### 2.2 Sample Collection

Five millilitres of venous blood samples were obtained from subjects during an 8-hour work shift, using a heparinised vacuum blood tube. Specimens were sent to the laboratory within a few hours and were processed immediately. About 15 to 20 mL of urine was obtained from subjects at the end of the work shift, using sterilised containers; it was stored at -20 ºC and analysed within one month.

### 2.3 Urinary TCA Analysis

Urinary TCA determination was performed by Gas Chromatography with Electron Capture Detector (GC-ECD), using a modified HS technique (Christensen, Rasmussen, & Koppen, 1988). A 1.0 mL sample of urine was added in a glass cap bottle, and then put in a headspace sampler that already set the optimum program. The sample was injected into the GC (HEWLETT PACKARD HP 6890) at 250 ºC, with Electron Capture Detector at 300 ºC (Column: HP-FFAP Polyethylene Glycol TPA, split mode 10:1) and oven temperature at 80 ºC for 12 min. The quantity of urinary TCA was analysed under the relative intensity of a chromatographic signal for 12 min. The limit of detection of TCA was 0.139 mg/L and the average coefficient of determination (r^2^) was 0.99972.

### 2.4 Blood Samples for Cytokinesis Block Micronucleus (CBMN) Assay

The blood samples were analysed using the CBMN assay according to Fenech et al. (2003). Lymphocyte culture was set up by adding 0.5 mL whole-heparinised blood in 5 mL of RPMI 1640 supplemented with 10% fetal bovine serum, 2% L-glutamine, 1% antibiotics (100 µg/mL penicillin and 100 µg/mL streptomycin) and 3% phytohemagglutinin. The cultures were incubated at 37 °C for 44 hrs, and then cytochalasin B (final concentration 3 mg/mL) was added for inducing binucleated cells. At 72 hr incubation, the cultures were harvested by centrifugation at 1000 rpm for 10 min. After centrifugation, cultures were briefly treated with a hypotonic solution (0.075 M KCl). For further processing, cells were then fixed twice in cold fixative solution (3:1 absolute methanol: glacial acetic acid) for 20 min at room temperature. After this step, the cell pellet was gently resuspended in a few drops of fresh fixative solution.

The slides were prepared for microscopic observation by carefully dropping the cell suspension on to clean, cold slides. Finally, the cells were air dried and stained with Giemsa. To determine the frequency of binucleated cells with micronuclei, 1,000 binucleated lymphocytes with clearly visible cytoplasm were scored under a light microscope (Olympus, USA) for each subject. Micronuclei were evaluated according to the criteria of Fenech et al. (2003), using 400X magnification for detection and 1,000X magnification for confirmation.

### 2.5 Environmental TCE Determination

The exposure to TCE was monitored during eight-hour work shifts by sampling the air in the breathing zone of the workers. During their shift, the workers wore diffusion samplers packed with activated charcoal on their collars, just under the mouth, to measure personal exposure concentrations of TCE. In addition to personal exposure assessment, active TCE sampling, for about 8 hrs at the position where the workers stayed most of the time, was performed with activated charcoal tubes and personal pumps (ALPHA-2 air sampler, Du Pont) at a flow rate of 200 mL/min. Measured concentrations of TCE were reported at eight-hour time-weighted averages (8 h TWAs). The samples were extracted with carbon disulfide and analysed by Gas Chromatography-Electron Capture Detector Headspace (GC-ECD-HS).

### 2.6 Statistics

The data were analysed using the SPSS 11.0 program for Windows (SPSS Inc., Chicago, IL, USA). The results were calculated as mean and standard error for each group. A student’s t-test for independent samples was used to detect the possible differences in the mean of micronucleus assay between the exposed and control groups. A correlation analysis was performed using Pearson’s correlation test; P < 0.05 and was considered statistically significant. The correlation between TCE exposure and subjective symptoms was evaluated by linear regression analysis.

## 3. Results

### 3.1 Characteristics of the Studied Population

The characteristics of the exposed group and non-exposed group (controls) are shown in Table 1. Both groups were characterised by gender, age and working time. A subjective symptom survey by self-administered questionnaire was also performed to evaluate any health problems, such as skin diseases, respiratory diseases, hypertension, allergies and haematological symptoms.

Skin symptoms, or dermatitis, was defined as an inflammation of the skin which varies from mild irritation to severe inflammation such as rash, large weeping or swelling. Respiratory symptoms refer to cough, frequent phlegm, sinusitis, bronchitis, irritation of throat, wheezing and breathlessness. Hypertension was identified by a level of blood pressure more than or equal to 140/90 mm of Hg, according to Thai Hypertension Society: Guidelines in the Treatment of Hypertension 2012. Allergies refer to the repeated hypersensitive reactions to chemical substances and to patients who reported suffering from skin or respiratory hypersensitivities. Information on haematological diseases of workers, such as anaemia, was obtained from the annual health examination record.

**Figure 1:**
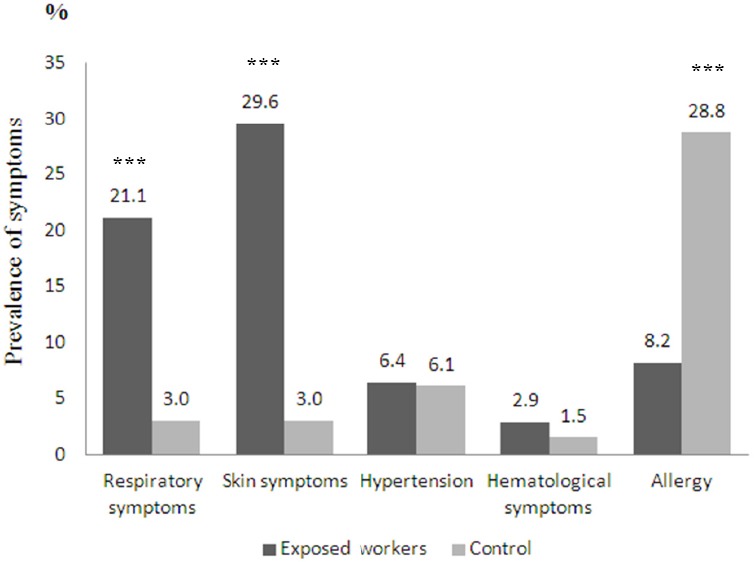
Comparison of prevalence of symptoms among exposed workers and control group Note: ***p=0.000

The exposed group comprised 171 workers with a mean age of 35.78 ± 9.54 (range 18-58 years). The mean age of the control group was 34.84 ± 12.96 (range 18-58 years). The result of statistical analysis revealed no significant difference of means of ages, smoking habits or alcohol consumption between exposed workers and control groups. The exposed group had a mean exposure of 11.13 ± 10.48 years, and hours of work per day were 10.77 ± 1.56. According to the information obtained from the questionnaire, 98.2% of exposed workers used a mask and gloves when exposed to chemical substances. The prevalence and odds ratio of symptoms among participants are presented in Table 2. Skin symptom was the most prevalent illness reported among the exposed workers (29.6%), followed by respiratory symptom (21.1%) and allergy (8.2%). In contrast, allergy was the most commonly reported symptom (28.8%) among control group (Figure 1). The exposed group had a significantly higher prevalence than the control group both in respiratory symptoms (OR = 9.61: 95% CI = 2.25-34.48) and in skin symptoms (OR = 11.76: 95% CI = 2.78-35.71). The prevalence of hypertension and haematological disorder was high among the exposed group compared with the control group, but not high enough to be statistically significant. In contrast, the exposed group had significantly lower prevalence rates of allergic response than the control group (OR = 0.22: 95% CI = 0.10-0.47).

**Table 2:** Prevalence and odds ratio of symptoms among TCE exposed group and control group

Symptoms	Exposed workers	Control	OR	95% CI	P-value
Respiratory symptoms	21.1% (36)	3.0% (2)	9.61	2.25-34.48	.000
Skin symptoms	29.6% (49)	3.0% (2)	11.76	2.78-35.71	.000
Hypertension	6.4% (11)	6.1% (4)	1.07	0.33-3.47	.916
Haematological symptoms	2.9% (5)	1.5% (1)	1.96	0.22-11.1	.536
Allergy	8.2% (14)	28.8% (19)	0.22	0.10-0.47	.000

### 3.2 Urinary TCA Determination and CBMN Assay

**Figure 2:**
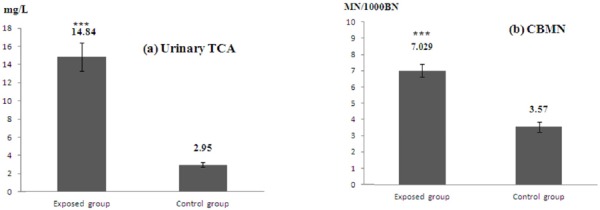
Comparison of (a) mean values of urinary TCA and (b) micronucleus by CBMN assay among exposed and control group

Note: *** p = 0.001

The level of exposure to TCE was assessed by a biomarker of exposure: urinary TCA. The mean concentration of TCA in the urine of exposed workers was 14.84 ± 1.62 mg/L, and control group was 2.95 ± 0.28 mg/L (Figure 2a). A significant difference between the exposed workers and controls concerning the TCE biomarker was observed (p = 0.001). Frequencies of lymphocytes with micronuclei in human peripheral blood cultures from both workers and controls were investigated, and the results are presented in Figure 2b. The mean MN/1000BN of the workers and the controls were 7.029 ± 0.39 and 3.57 ± 0.31 respectively. The result showed that the exposed workers revealed a significant induction of MN when compared with the controls (p = 0.001).

### 3.3 Environmental TCE Determination

Time-weighted average (TWA) concentrations of personal TCE exposure on the factory lines where TCE was used are shown in Table 3. The mean value for exposure of TCE in the air of the workplaces of employees was 27.83 ± 6.02 ppm, which is below the current US Occupational Safety and Health Administration permissible exposure limit (100 ppm 8h TWA). The average ceiling value was 21.05 ± 13.13 ppm. In addition, the ceiling concentration was lower than the American Conference of Industrial Hygienists reference value (200 ppm).

**Table 3:** Workplace TCE concentrations

Sample site	Rate of air suction (ml/min)	TCE (ppm)	

		TWA	Ceiling
Baking room	200	32.08	11.76
Washing room	200	23.57	30.33
Mean		27.83±6.02	21.05±13.13

In order to search for an association between urinary TCA and independent variables such as age, symptoms, and duration of work (year) among exposed subjects, a Pearson correlation was performed. A significant association was observed between urinary TCA and duration of work (years) (r = 1.54, p = 0.004), although no significant association was seen with other independent variables (Table 4).

**Table 4:** The correlation coefficients (r) between urine TCA level and independent variable among study subjects using Pearson correlation

Parameters	Urinary TCA	

	R	P
Age	.102	.185
Personal protective equipments	-.035	.647
Duration of work (years)	1.54	.004*
Respiratory symptoms	-.074	.335
Skin symptoms	-.066	.389
Hypertension	-.005	.951
Haematological symptoms	-.054	.479
Allergy	-.037	.633

* Correlation is significant at the 0.01 level (2-tailed)

A Pearson’s coefficient correlation was also carried out to investigate the associations regarding age, gender, smoking status, alcohol consumption, duration of work and urinary TCA in determining the frequency of MN in exposed workers. Correlation analyses pointed to the association of urinary TCA with the level of MN among the exposed workers, where a significant positive associated with MN frequency was noticed (r = .285, p < 0.001) (Table 5). In addition, of the other confounding factors studied, no significant association could be seen.

**Table 5:** The correlation coefficients (r) between MN frequency and independent variable among study subjects using Pearson correlation

Parameters	MN frequency	

	R	P
Gender	-.003	.937
Age	.071	.353
Smoking status	.006	.934
Alcohol consumption	-.019	.803
Personal protective equipments	-.018	.812
Duration of work (years)	.058	.450
Urinary TCA	.285	.000*

* Correlation is significant at the 0.01 level (2-tailed).

The results of the multiple linear regression analysis revealed a significant relationship between DNA damage (MN frequency) and urinary TCA level in exposed workers (p < 0.001). However, Table 6 illustrates that the R-squared and the adjusted R-squared values of the regression results for predicting the DNA damage (0.085 and 0.058) show a weak correlation between the predictive factors and MN frequency.

**Table 6:** Multiple linear regression analysis between micronucleus frequency and other factors in exposed workers

Factors	Coefficient	Std Error	T test	p-value
Constant	4.159	2.430	1.711	.089
Age	.119	.077	.828	.409
Duration of work (years)	-.085	.070	-.589	.557
Smoking status	.029	.338	.338	.736
Alcohol consumption	-.025	.493	-.295	.768
Urinary TCA	.285	.017	3.774	.000
R Squared	.085			
Adj R Sq	.058			
F Value	3.081			

## 4. Discussion and Conclusion

In an occupational setting, workers directly involved in manufacture, especially those working in the degreasing process, may be exposed to TCE at higher levels than the general population. A high level of TCE has been detected in settings that use TCE as an industrial solvent. The main avenue of TCE exposure in industrial workers is through inhalation. In our study, a CBMN test was used for surveillance of chromosomal damage in the peripheral lymphocytes of workers occupationally exposed to TCE. The permissible exposure limit for workers exposed to TCE, recommended by the Occupational Safety and Health Administration, is 100 ppm for 8-hour TWA (Occupational Safety and Health Administration [OSHA], 2012). In this study, TCE levels in the workplace air were 27.83 ± 6.02 ppm. This did not exceed the level of TWA permission (< 100 ppm) in factories for either the personal breathing zone or the area monitors.

Our results confirmed the study of Ruder (2006), who reported that more than 1.5 million workers were being exposed to chlorinated solvent every year, and that the mean occupational exposure levels of the workers was 59 ppm or higher, depending on the job (Gold, De Roos, Waters, & Stewart, 2008). Although most workers were exposed to TCE levels below the current permissible exposure limit of 100 ppm recommended by OSHA, some studies revealed evidence that TCE is immunotoxic at low exposure levels (Zhang et al., 2013). As indicated by several studies on occupational epidemiologic surveys, exposure is typically characterised by urinary biomarker data (Bolt, Lammert, Selinski, & Brüning, 2004; Green et al., 2004). Many studies have shown that TCA in urine is the preferred biological index to monitor TCE exposures. Imbriani, Niu, Negri, and Ghittori (2001) observed a linear correlation between the time-weighted average exposure to TCE and the concentrations of urinary TCA. In the present study, the evidence of exposure, expressed as the presence of urinary TCA, was found in all 171 members of the exposed group. The mean concentration of TCA of exposed workers was 14.84 ± 1.62 mg/L, which showed a significantly higher level than that found in controls (2.95 ± 0.28 mg/L). The result is in good agreement with other studies such as Bakke, Stewart, and Waters (2007), who reported that the mean concentration of urinary TCA in exposed workers was 63 umol/L (10.3 mg/L). The biological monitoring study of workers in various occupations, conducted by the Finnish Institute of Occupational Health, demonstrated that the overall median concentrations of TCA in urine were 10.3 mg/L for women and 7.8 mg/L for men (Anttila, Pukkala, Sallmén, Hernberg, & Hemminki, 1995). The National Occupational Exposure Survey, conducted from 1981 to 1983, estimated that 401,373 workers at 23,225 facilities were potentially exposed to trichloroethylene (National Institute for Occupational Safety and Health [NIOSH], 1990).

During health hazard investigations by the National Institute for Occupational Safety and Health, mean occupational exposure concentrations of trichloroethylene in the air, ranging from 1.3 mg/m^3^ to 1,084 mg/m^3^, were reported. In addition, Green et al. (2004) reported that the average TCA in urine of exposed workers in an occupational medical field was 32 ppm, with an average length of exposure of 4.1 years (total range: 1 to 20 years). The authors mentioned a relationship of decreased Vitamin B12 levels with long-term TCE exposure. Vitamin B12 deficiency leads to excess formic acid and methylmalonic acid in urine, resulting in nephrotoxicity by cellular acidosis. However, the clinical relevance of nephrotoxic effects has not been reported.

In our study, the average duration of occupational exposure to TCE was 11.13 ± 10.48 years, ranging from less than one year to 20 years. The results indicated that long-term exposure to TCE may at least contribute to the higher frequency of MN in exposed workers when compared to controls. TCE and a number of its metabolites have been evaluated by many genotoxic assays, and proved by several studies that they are responsible for the toxicity to cells and specific organs (Lash, Fisher, Lipscomb, & Parker, 2000a). The evidence of high concentrations of TCE metabolites that bind to DNA and cause strand breaks has been shown in the study of TCE genotoxicity (Tabrez & Ahmad, 2009). Current knowledge exists on the effect of TCE metabolites on tumour formation, which can be explained by the induction of chromosome or genome changes, such as hypomethylation by these metabolites (Shiao, 2009). Vermeulen et al. (2012) found that low-level exposure to TCE was associated with changes in a nephrotoxicity biomarker – kidney injury molecule-1 (KIM-1) – and was possibly linked to an increased incidence of renal cancer. Varshney, Chandra, Chauhan and Goel (2013) also reported that TCE metabolites such as TCA, DCA and TCEOH induced MN production in lymphocytes, which suggests their genotoxic potential.

In our study, we found a significant increase in MN frequency in exposed workers. This finding is similar to other studies reporting a significant increase in MN frequency with occupational exposure to TCE (Gentile et al., 2012; Sudha et al., 2011; Wojda, Zietkiewicz, & Witt, 2007). The differences in MN frequency among various TCE-exposed workers have also been observed in previous studies by Rozgaj, Kašuba, and Jazbec (2001). As reported by many researchers, an increase in the frequency of MN has been found in CHO-K1 cells and lymphocytes after exposure to vaporous TCE in vitro (e.g., Wang, Chen, Tsai, Sung, & Huang, 2001). In addition, Cheng et al. (2012) reported the increase of nuclear bud formation in lymphocytes of exposed workers who had a level of TCA higher than 50.0 mg/L or had a longer occupational history. Similarly, Hu et al. (2008) also found that DNA damage in peripheral lymphocytes of TCE workers increased, using the single cell gel electrophoresis method. The significant increase of MN frequencies in the cells exposed to TCE clearly indicates that TCE can induce chromosome damage. In our study, the association between MN frequency and exposure to TCE (as indicated by TCA in urine) was observed. Recent studies provide biological plausibility of the association between occupational TCE exposure and risk of renal cancer (Moore et al., 2010).

Inhalation is the main route of exposure for TCE (Bakke et al., 2007). Once vaporous TCE enters into the respiratory tract, it can irritate both the upper and lower respiratory tract. The most common symptoms found in workers exposed to TCE were respiratory symptoms, central nervous system toxicity and skin irritation (Bale, Barone, Scott, & Cooper, 2011). In our study, we found that the predominant abnormal manifestations among these exposed workers were skin and respiratory symptoms. The incidence of skin symptoms in workers was noted by Xu et al. (2009). Similar symptoms were found by Strong et al. (2004) among workers exposed to TCE. In addition, various studies have demonstrated that inhalation of this vaporous solvent may be linked to an increase in prevalence of allergic diseases. It is now known that inhaled allergens can enhance the airway response, resulting in exacerbation of asthma. Davis, Laszlo, Wu, Sapp and Cusack (2005) reported an association between TCE exposure and an increased risk of hypertension. Raşcu, Bucur, Naghi, and Drăghici. (2003) demonstrated that TCE can induce systemic respiratory syndrome. In our study, urinary TCA was determined as a biomarker of exposure, and micronuclei in lymphocytes were counted as an indicator of chromosomal and DNA damage, measured in terms of a CBMN test. A significant positive correlation was found between micronucleus frequency and urinary TCA concentration (r = 0.258, p < 0.001) of exposed workers, indicating that DNA damage is directly proportional to TCE exposure. In addition, our findings on the urinary TCA were significantly higher in exposed workers (14.84 ± 1.62 mg/L) than control groups (2.95 ± 0.28 mg/L) (p < 0.001). This could be due to the fact that the workers were directly exposed to TCE in their workplace. A similar result was observed in dry-cleaning workers exposed to perchloroethylene (Everatt, Slapšytė, Mierauskienė, Dedonytė, & Bakienė, 2013). A recent report by Kleinsorge et al. (2011) found a significant association between duration of work and MN frequency in buccal cells. The results highlight the possible influence of exposure time on DNA damage. In addition, according to multiple linear regression analysis, only urinary TCA presented a dominant tendency (p < 0.001) to describe the increase in genetic damage. This result suggests that TCE is associated with genotoxic effects.

In conclusion, workers at the clock manufacturing factory had significant exposure to TCE in their workplace. The level of chromosomal damage measured by CBMN was significantly higher in the exposed workers compared to the controls. The present study also revealed an association between TCE exposure and chromosomal damage (increased MN frequency). The cytogenetic damage in workers exposed to TCE was associated with the concentration of TCE exposure. This study supports the need to educate those who work with potentially hazardous materials about adverse effects, and highlight the importance of using protective equipment.
